# Symbiont Gene Expression Predicts Insect Host's Response to High Temperatures

**DOI:** 10.1111/mec.70154

**Published:** 2025-10-28

**Authors:** Patrick T. Stillson, Sheina B. Sim, Renee L. Corpuz, Alison Ravenscraft

**Affiliations:** ^1^ Department of Biology University of Texas at Arlington Arlington Texas USA; ^2^ USDA‐ARS Daniel K. Inouye US Pacific Basin Agricultural Research Center Hilo Hawaii USA

**Keywords:** *Caballeronia*, gene expression, heat shock, Heteroptera, *Leptoglossus phyllopus*, symbiosis, thermal stress

## Abstract

Microbial symbionts play crucial roles in host nutrition, defence, and detoxification. However, host‐symbiont interactions are context‐dependent, and environmental stressors can disrupt these benefits. Diverse hosts, including corals, insects and leguminous plants, have been shown to suffer under thermal stress due to the negative impact of high temperatures on their symbionts. This failure is often linked to a symbiont's poor transcriptional regulation of heat shock genes, causing vulnerability at high temperatures. In the bug‐*Caballeronia* model system, insect performance at elevated temperatures varies based on the hosted symbiont species. Here, we explore the underlying mechanisms that drive this variation using comparative metatranscriptomics and two symbionts with contrasting host outcomes at high temperatures. We evaluated both host and symbiont transcriptional responses to elevated temperature, testing the hypothesis that symbionts conferring improved host outcomes at high temperatures will have more upregulated heat shock genes under thermal stress compared to those conferring worse host outcomes. Our findings reveal that host transcription did not change with different symbionts but rather only at different temperatures. Furthermore, symbionts had distinct gene expression profiles across temperatures. At 36°C, the heat‐resistant symbiont not only increased expression of heat shock genes but surprisingly upregulated flagellar genes, which are normally turned off during symbiosis. This suggests that symbiont, not host, transcription underlies host benefits at low versus high temperatures and ultimately furthers our understanding of context dependence in the outcomes of symbiotic associations.

## 1 Introduction

Most multicellular organisms utilise microbial symbionts for nutrient supplementation, parasite protection, chemical detoxification, or other services (Engel and Moran 2013; Kikuchi et al. 2012; Moran 2007; Oliver et al. 2003). Although these interactions are often important for host survival, they are context dependent (Bronstein 1994; Hoeksema and Bruna 2015), with the degree of benefits provided to each partner shifting as environmental conditions change. Under stressful conditions, symbioses may even break down, turning from mutualistic to antagonistic (Bronstein 2001). Therefore, understanding how microbial symbionts respond to environmental pressures can reveal how symbioses are maintained or degrade under stress.

At high temperatures, outcomes for a host are often reliant upon the survival of its symbiont. If heat disables or kills the symbiont, the host will typically suffer from nutrient deficiencies, decreased growth, lower fecundity, and increased mortality (Dunbar et al. 2007; Kikuchi et al. 2016; Oakley and Davy 2018). This thermal vulnerability in the symbiont is often associated with low expression of heat shock‐associated genes that maintain protein and cellular stability at high temperatures. The heat shock pathway starts with transcriptional regulatory proteins that initiate the heat shock cascade, activating various proteins including molecular chaperones that repair damaged and misfolded proteins, and proteases that remove damaged proteins and polypeptides from the cell by breaking them down into their base components (reviewed in Roncarati and Scarlato 2017). These heat shock genes are utilised by both prokaryotic and eukaryotic microbial symbionts in response to various stressors (i.e., DNA damage, heat shock, oxidative stress, etc.), but their roles in the thermal stress response are particularly well understood (Rangel 2011).

Many studies of temperature dependence in symbiotic interactions have investigated hosts with vertically transmitted symbionts (microbes that are transmitted directly from parent to offspring). In these systems, the microbe often undergoes a phenomenon called genome reduction. Because the host typically provides the symbiont with a stable environment, many of the symbiont's genes go unused, leading to an accumulation of mutations and loss of gene function. The symbiont genome shrinks, ultimately retaining only those genes required by the host or symbiont for survival (Bennett and Moran 2015; McCutcheon and Moran 2012). Symbiont heat shock genes are frequently disabled or deleted, and as a result, vertically transmitted symbionts are often vulnerable to high temperature.

For example, high temperatures reduce or even eliminate the *Buchnera* symbiont from aphid hosts, resulting in reduced fecundity and survival (Dunbar et al. 2007; Zhang et al. 2019). However, aphids from hotter regions (Arizona, USA) have symbionts with higher thermal tolerances than those found in cooler regions. These heat‐adapted aphid symbionts have a mutation in the promoter of the heat shock gene *ibpA*, increasing the expression of this gene and improving symbiont performance under heat stress (Dunbar et al. 2007; Zhang et al. 2019). This mutation is absent in species dwelling in cooler environments, making them vulnerable to high temperatures (Dunbar et al. 2007) and restricting the host's habitable range to regions in which the symbiont can survive. In aphids, stinkbugs and weevils, symbiont genome reduction has likely contributed to the reduction in the symbiont's thermal tolerance through the loss of heat shock genes. These genes have largely lost their functionality in the symbionts either through decreased expression or gene deletion (Anbutsu et al. 2017; Bennett and Moran 2015; Kikuchi et al. 2016). This can limit host survival under extreme temperatures, such as abnormal climatic conditions or hosts moving outside their normal range to hotter regions.

However, if a host depends on a horizontally acquired symbiont that experiences a free‐living stage, the symbiosis may be less vulnerable to high temperature for at least two reasons. First, the host can potentially associate with a diverse and functionally heterogeneous environmental pool of partners. Second, symbionts with a free‐living stage will have larger genomes because they have not undergone genome reduction and therefore may have a more robust heat shock response. For example, the thermally tolerant rhizobial symbionts of legumes express heat shock genes DnaK and GroESL at higher levels compared to heat‐sensitive strains (Alexandre and Oliveira 2011). This increase in heat shock expression is essential for the initiation and maintenance of the symbiosis, as free‐living rhizobia cannot colonise their hosts if they are unable to tolerate high thermal stress (Alexandre and Oliveira 2013). This is also observed in the algal symbiont of corals, with heat shock gene expression increasing as part of the thermal stress response in both symbiotic and free‐living cells (Leggat et al. 2011), with more thermally tolerant symbiont species better able to survive high temperature events and acting as better symbionts under high temperature conditions (Berkelmans and Van Oppen 2006).

The bug‐*Caballeronia* symbiosis is a promising model system to investigate how environmentally acquired symbionts affect host survival and development under thermal stress (Stillson et al. 2025). In this symbiosis, *Caballeronia* provides thousands of species of true bugs with essential amino acids and B vitamins (Ohbayashi et al. 2019). Hosts must acquire the symbiont from the environment each generation during the second instar (developmental stage) (Itoh et al. 2019; Kikuchi et al. 2007), or suffer high mortality, developmental delays, and reduced body size (Acevedo et al. 2021; Hunter et al. 2022; Itoh et al. 2019). Hosts can associate with symbiont species from across the *Caballeronia* genus (Garcia et al. 2014; Ravenscraft et al. 2020), with symbionts having little to no host specialisation (Stoy et al. 2023) and usually conferring equivalent host outcomes under benign conditions (Acevedo et al. 2021; Hunter et al. 2022). However, we previously found that under thermal stress, there are variations in symbiont‐dependent host outcomes (Stillson et al. 2025). In Eastern leaffooted bugs ( *Leptoglossus phyllopus* , Hemiptera: Coreidae) colonised by one of six species of *Caballeronia*, different symbionts conferred the best host outcomes (lower mortality, faster development, higher weight) at cooler versus warmer temperatures. Outcomes diverged drastically as temperatures increased (> 28°C), with some symbionts imposing severe costs under thermal stress.

**FIGURE 1:**
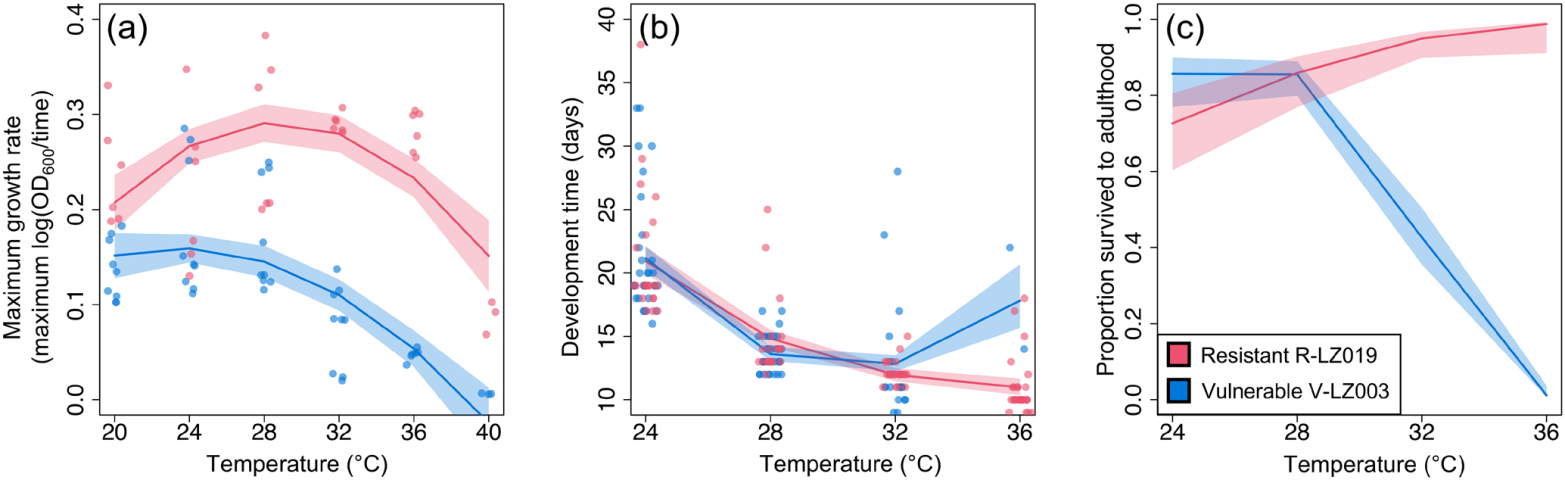
(a) Symbiont in vitro maximum growth rates for the heat‐resistant symbiont and heat vulnerable symbiont and (b) development time and (c) survival of host insects inoculated with one of the two isolates and reared at different constant temperatures (modified from Stillson et al. 2025).

Here we investigate the underlying mechanisms that may explain temperature‐dependent variation in this symbiosis under thermal stress. Two *Caballeronia* symbionts (V‐LZ003 and R‐LZ019) were selected from this prior work based on differences in host outcomes at high temperatures. While reared at a cool 24°C, insects provided with either symbiont strain developed at similar rates and had comparable survival, but at a hot 36°C, insects with the heat‐resistant symbiont outperformed insects with the heat‐vulnerable symbiont (Figure 1). By analysing in vivo host and symbiont transcriptional variation between these two symbiont species, we aim to elucidate genetic mechanisms that may underlie this temperature‐driven variation. Our results reveal that host transcription does not change with different symbionts, but rather symbiont transcriptional differences predict host outcomes. As expected, the resistant symbiont upregulated more heat shock genes at high temperature compared to the vulnerable symbiont. Surprisingly, while *Caballeronia* symbionts are known to be non‐motile in vivo, the resistant symbiont also upregulated numerous flagellar genes under thermal stress conditions.

## 2 Materials and Methods

### 2.1 Sequencing, Assembling, and Annotating the Host Genome

To facilitate analysis of the host transcriptome, we sequenced and annotated the *L. phyllopus* genome. One male and one female *L. phyllopus* from our lab colony were shipped on dry ice from the University of Texas at Arlington to the USDA‐ARS Tropical Pest Genetics and Molecular Biology Research Unit in Hilo, Hawaii. Upon receipt, samples were stored at –80°C until DNA extraction. Each insect was sequenced with both HiFi and HiC approaches to produce long and short reads (respectively) that were combined to produce final genome assemblies for both the male and female insects.

#### 2.1.1 PacBio HiFi Library Preparation

Each insect's head, thorax, and half of the abdomen were ground into a powder. (The other half of the abdomen was reserved for HiC library preparation, described below.) A Qiagen MagAttract HMW DNA Kit (Qiagen, Germany) was used to isolate high‐molecular weight DNA followed by quantification using a Qubit dsDNA High Sensitivity (HS) assay (ThermoFisher Scientific, Massachusetts, USA) measured using the fluorometer function of a DS‐11 Spectrophotometer and Fluorometer (DeNovix Inc., Delaware, USA). We evaluated DNA purity using ratios of absorbance at 260, 280 and 230 nm measured by the spectrophotometer function of the DS‐11 Spectrophotometer and Fluorometer. After DNA isolation, quantification, and purity assessment, genomic DNA was sheared using a Diagenode Megaruptor 3 (Denville, New Jersey, USA) to a mean sheared size of 15 kb, and we determined the final size distribution of the genomic DNA using a Femto Pulse System (Agilent, Iowa, USA). For both the male and female samples, we prepared a SMRTbell library for PacBio using the SMRTBell Express Template Prep Kit 2.0 (PacBio, CA, USA) and sequenced both using one Revio SMRT Cell for each library. After sequencing, we used the PacBio Short Read Eliminator (SRE) XS Kit to deplete the remaining library of fragments shorter than 10 kb. Following SRE treatment, both libraries were sequenced on another Revio SMRT Cell. For both RevioSMRT Cell outputs, SMRTLink v12.0.0.177059 was used to call circular consensus reads from the raw subread data.

#### 2.1.2 HiC Library Preparation

Simultaneous to the PacBio HiFi sequencing pipeline (above), we prepared a short‐read HiC library with the remaining tissue from each insect. We began the short‐read HiC library preparation by first cross‐linking the tissue with 2% formaldehyde and digesting using the enzymes DdeI and DpnII which was followed by proximity ligation and DNA isolation. We prepared the sequencing library using the NEB Next Ultra II DNA Library Prep Kit followed by the Element Biosciences Adept Library Prep Kit. We sequenced the resulting HiC libraries on one partial flow cell of an Element Biosciences Aviti System.

#### 2.1.3 Sequence Data Processing and Assembly

After the HiFi sequencing, but prior to contig assembly, we removed the PacBio Blunt Adapter contaminated sequencing artifacts using HiFiAdapterFilt v3.0.0 (Sim et al. 2022). After adaptor removal, we assembled the genome into contigs using HiFiASM v0.19.3‐r572 (Cheng et al. 2021). Following contig assembly, we removed duplicate contigs using PurgeDups (Guan et al. 2020). We mapped the paired‐end HiC reads to the duplicate purged contig assembly and used the YaHS v1.1 pipeline (Zhou et al. 2023) to create a HiC contact map which we manually edited using Juicebox v1.11 (Durand et al. 2016).

To evaluate the HiFi contig and HiC scaffolded assemblies, we used the NCBI FCS‐GX software and Blobtools2 v4.1.5 (Challis et al. 2020) and used nucleotide BLAST+ (Camacho et al. 2009) on the NCBI NT database (accessed 2022‐02‐14) and Diamond BLAST (Buchfink et al. 2015) on the UniProt protein database (accessed 2020‐03) to identify non‐arthropod contigs. Once identified, we removed non‐arthropod contigs from the assembly after scaffolding. We used MitoHiFi v3.0.0 (Uliano‐Silva et al. 2023) and the representative mitochondrial genome from *Cloresmus pulchellus* on NCBI (RefSeq accession: NC_042806.1) to identify and annotate all mitochondrial contigs from the contig assembly prior to duplicate contig removal. All but one mitochondrial contig was removed from the final assembly. To assess genome completeness, we used the BUSCO v5.2.2 pipeline (Manni et al. 2021) to perform a *de novo* annotation of a benchmark of universal single copy orthologs from the Hemiptera ortholog dataset on the contig assembly, the duplicate purged contig assembly, and the HiC scaffold assembly.

#### 2.1.4 Annotation

To annotate the *L. phyllopus* genome, we masked the repetitive elements using RepeatModeler 2.0.5 (Flynn et al. 2020) and RepeatMasker v4.1.5 (Smit et al. 2013), and mapped trimmed RNA‐seq reads (SAMN40545253) to the masked genome using HISAT2 v2.2.1 (Kim et al. 2015). The output was converted to a sorted BAM file using Samtools sort v2.0.5 (Danecek et al. 2021), which was used to annotate the genome with BRAKER3 v3.0.8 (Brůna et al. 2021). The BRAKER3 program annotates genomes using closely related species protein sequences as well as using RNA transcripts, so for this we acquired an arthropod protein dataset from OrthoDB v11.0 (Kuznetsov et al. 2023) to use in the annotation process.

### 2.2 Symbiont Species Identification

Using the two symbiont genomes (accessions: heat‐vulnerable symbiont V‐LZ003—GCF_031451465.1; heat‐resistant symbiont R‐LZ019—GCF_031450825.1), we calculated the average nucleotide identity between the symbionts to determine whether they were the same species (http://enve‐omics.ce.gatech.edu/ani/) (Rodriguez‐R and Konstantinidis 2016). We found that the symbionts had an ANI score of 82.23%, indicating that they are closely related but not the same species; therefore, we opted to evaluate the transcriptional expression levels of each symbiont separately (Goris et al. 2007).

### 2.3 Insect Rearing and Symbiont Provisioning Procedure

Following previously used methods (Stillson et al. 2025), host insects were acquired from our laboratory colony of *L. phyllopus*, maintained at 28°C with a 16L:8D light cycle. Insects were fed bush bean plants ( *Phaseolus vulgaris* ) and raw Spanish peanuts.

Freshly laid eggs were collected and transferred into screened plexiglass boxes (11.33 cm × 11.33 cm × 4 cm; hereafter referred to as cages). Cages contained cowpea cuttings ( *Vigna unguiculata* ), a cotton‐stoppered vial filled with 0.5% vitamin C water (L‐ascorbic acid), and two peanuts. Water for plants was refilled as necessary, vitamin C vials were replaced weekly, and peanuts were replaced every 2 weeks.

At second instar, insects were provided with one of the two *Caballeronia* symbionts (heat‐vulnerable symbiont V‐LZ003 or heat‐resistant symbiont R‐LZ019). *Caballeronia* suspension was provided in a cotton‐stoppered 1.7 mL microcentrifuge tube, while the plant and vitamin C were removed to ensure the nymphs drank the cell suspension. The cell suspension was prepared by taking 1 mL of overnight *Caballeronia* culture grown in yeast‐glucose broth (approximately 1.0 OD) (Kikuchi et al. 2011), pelleting the cells, and resuspending the pellet in 1 mL of 0.5% vitamin C water. This suspension was replaced every 24 h for 4 days, after which the plant and vitamin C water were returned. Eggs and all nymphs through third instar were maintained in Percival chambers (Percival Scientific, Iowa, USA) set to 28°C, 16 L:8D light cycle, with an average relative humidity of 50%.

Upon moulting to third instar, nymphs were transferred to three different thermal treatment groups, with an average of five insects per cage. These cages were placed into Percival chambers set at 24°C, 30°C and 36°C, with a 16L:8D light cycle and 50% average relative humidity. When insects were in the middle of the fourth instar stage, they were placed singly into 1.7 mL microcentrifuge tubes and flash frozen in liquid nitrogen. Then 1 mL of RNAlater was added to the tube, and it was stored at −20°C.

### 2.4 RNA Extraction and Sequencing

Total RNA (*n* = 3 per temperature × symbiont treatment) was extracted following the Promega SV total RNA isolation system protocols for small tissue samples (Madison, WI, USA). To maximise sequencing of symbiont RNA, each replicate consisted of 10 dissected symbiotic organs (the M4 region of the insect gut), which were pooled to reduce individual‐level variation and increase the representativeness of each sample. Pooling organs from multiple individuals was specifically chosen to mitigate biological variability and strengthen the robustness of each sample. The organs were determined to be colonised if they appeared to be swollen and opaque, which is indicative of colonisation. From this, we produced 18 total samples (2 symbionts × 3 temperatures × 3 replicates).

Total RNA was sequenced by Novogene (Beijing, China), using Novogene's dual‐seq library preparation protocol. Sequencing was performed on a NovaSeq PE150 (Illumina, San Diego, CA, USA) and 10 Gb of raw reads was acquired for each sample.

### 2.5 Mapping Reads

Reads were trimmed with Cutadapt v.4.4 (Martin 2011) to remove the adapter sequences and to remove reads with *N* > 10%, low quality scores, or shorter than 20 bp. After the cleanup, host reads were mapped to the male *L. phyllopus* genome using BWA‐MEM v.0.7.17.2 (Li 2013) with the default settings. Afterwards, the remaining unmapped reads were mapped to the corresponding symbiont genome based on the treatment group. Mapped reads were assigned to genes and counted with featureCounts v.2.0.3 (Liao et al. 2014) using the default settings.

### 2.6 Statistical Analysis

Multidimensional scaling (MDS) was performed on variance‐stabilised data using ‘varianceStabilizingTransformation’ (package = ‘DESeq2’) (Love et al. 2014) to compare each symbiont's gene expression profile across temperatures. No filtering of lowly expressed genes or removal of sample outliers was performed prior to this step; all genes were retained to preserve the full expression landscape for exploratory analyses. For differential expression analysis, we used the voom‐limma method (Law et al. 2014; Ritchie et al. 2015) implemented via Degust (Powell 2015), which applies voom normalisation and linear modelling. Genes were filtered to include only those with a minimum count‐per‐million (CPM) of 0.5 in at least two samples. We subsequently used log2‐fold change **≥ |**1**|** from this analysis for gene ontology (GO) enrichment using the R package ‘GO_MWU’ (https://github.com/z0on/GO_MWU) (Wright et al. 2015). Except for the ANI and Degust analyses, all statistical procedures were performed in R v4.3.2 (R Core Team 2025).

## 3 Results

### 3.1 Insect Genome Sequencing and Assembly Statistics

The first Revio SMRT Cell yielded 4.9 million HiFi reads with a total yield of 57.39 Gb and an N50 length of 14.9 kb, and the second Revio SMRT Cell yielded 5.6 million HiFi reads with a total yield of 71.17 Gb and an N50 length of 14.6 kb. In total, the genome was sequenced to about 84× coverage and was assembled into 1990 contigs. The HiC library was sequenced to about 10× coverage of the genome or about 60 million read pairs. After HiC assembly, the final genome was a total size of 1.67 Gb in 9 autosomes, one X chromosome, 131 unplaced contigs, and the complete mitochondrial genome (Figure S1, Table S1). After genome assembly, we evaluated genome completeness using a Benchmark of Single Copy Orthologs (BUSCOs) in the Eukaryote and Hemiptera v.10 databases, revealing a completeness score of 99.6% and 98.9%, respectively (Table S2). Taxonomic assignment of the contigs in the assembly using the Blobtools v.2 pipeline, BLAST+ to the NCBI nucleotide database, and Diamond BLAST to the Uniprot protein database revealed 170 non‐arthropod contigs that were removed from the assembly prior to NCBI submission.

We identified 59.68% of the *L. phyllopus* genome as repetitive elements. These regions were masked, and we annotated the masked genome using the BRAKER3 pipeline. This resulted in 11,843 annotated genes, of which 4810 (40.61%) have functional annotations. An estimate of annotation completeness using BUSCOs in the Eukaryote and Hemiptera databases revealed a completeness score of 97.6% and 93.7%, respectively (Table S3).

### 3.2 Transcript Statistics

Reference transcriptomes were constructed for the host by mapping the transcripts against the *L. phyllopus* genome for all 18 samples. There was an average of 77.7 (SD = 10.2) million host reads per sample (83.31% mapping efficiency). The remaining reads for each sample were then mapped against the corresponding symbiont genome for that treatment group. There was an average of 4.5 (SD = 0.5) and 1.3 (SD = 0.5) million reads mapped to the bacterial reference genomes per sample for the vulnerable and resistant symbionts, respectively (Table S4).

### 3.3 Host Gene Regulation Under Thermal Stress

Before evaluating the symbiont transcriptional response, we first evaluated the host gut transcripts to determine whether host gene expression could provide an explanation as to why different symbionts elicit variable responses in host outcomes. We performed differential expression analysis using a model with symbiont identity and temperature as main effects (FDR corrected *p* < 0.01, with a log2‐fold change ≥ |1|). An interaction model (~symbiont × temperature) failed to run, likely due to limited statistical power from small sample sizes within each treatment combination. Therefore, only the additive model was retained.

MDS plots showed that host gene expression patterns at 24°C and 30°C were similar, while 36°C samples were distinct. Similar clustering was later observed for the symbiont transcriptomes as well (see below). Because the 24°C and 30°C temperatures did not differ substantially for either the host or the symbionts, we retained the 30°C group in early analyses to illustrate these similarities but excluded it from final differential expression tests to focus on the thermal contrast between low (24°C) and high (36°C) temperature conditions.

**FIGURE 2:**
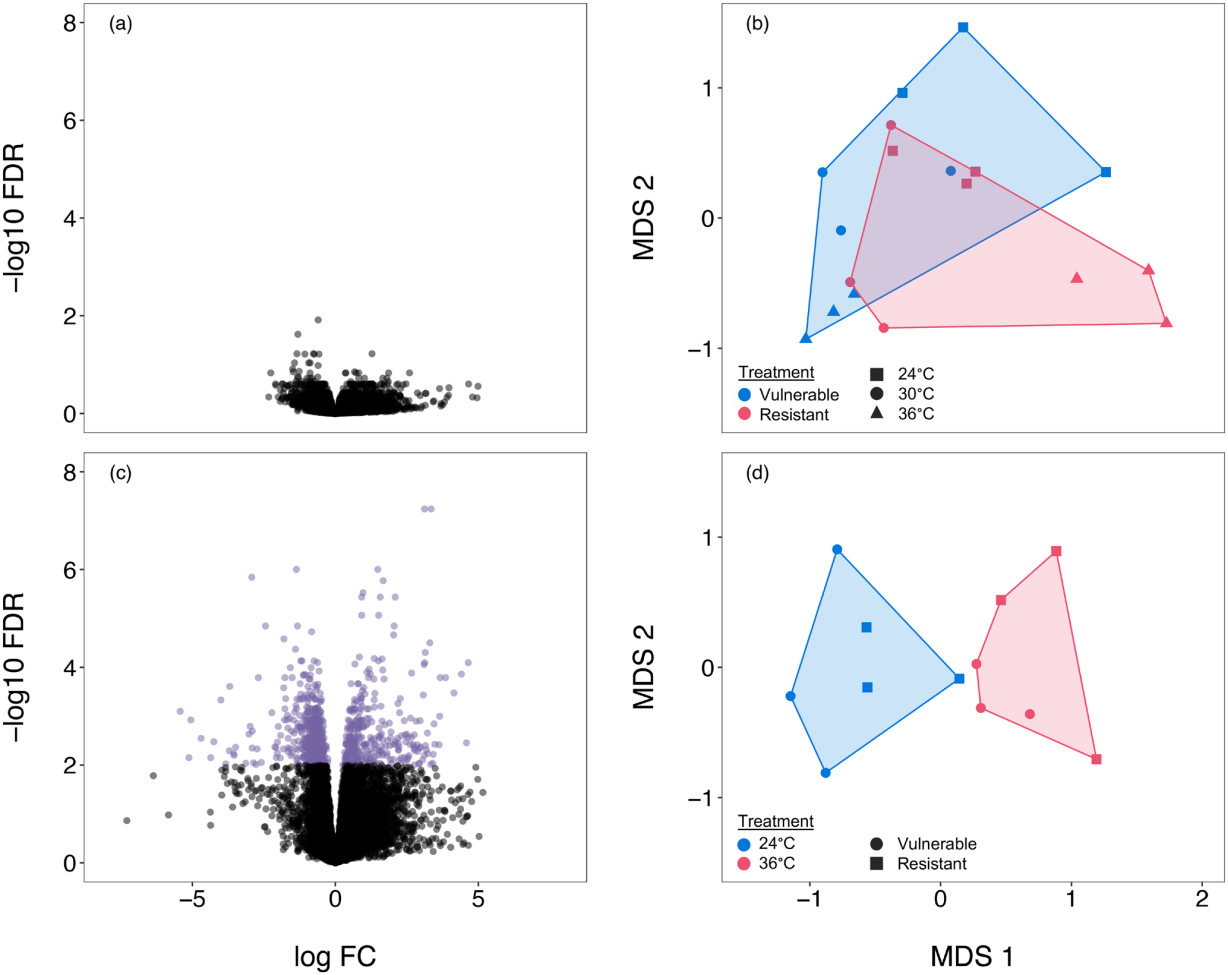
Host transcriptional variation depicted with volcano plots, with coloured points indicating differentially expressed transcripts by pairwise comparison (FDR‐corrected *p* ≤ 0.01, greater than log2‐fold change ≥ |1**|**) for (a) vulnerable–resistant symbiont comparison and (c) 24°C–36°C thermal comparison. Multidimensional scaling showing ordination of replicates for each of the treatment groups of (b) vulnerable—resistant and (d) 24°C–36°C.

We found that host gene expression remained the same regardless of symbiont identity, with zero differentially expressed genes detected (Table S5, Figure 2a,b), while temperature had a significant effect. When comparing hosts reared at 24°C and 36°C, 272 genes were differentially expressed, of which 100 genes were annotated (Figure 2c,d). Among these temperature‐associated genes, 35% were annotated with GO terms related to biological processes (e.g., stress response, protein folding) and 12% to cellular components (e.g., membranes, organelles). Metabolic processes, a subset of biological processes, accounted for 10% of all host temperature‐associated genes. Notably, none were annotated with GO terms directly associated with canonical heat stress functions (e.g., heat shock proteins).

We subsequently evaluated whether the expression of 76 host chaperone proteins or 133 immune genes (identified in the related species *Anasa tristis* (Mendiola et al. 2024)) changed as temperatures increased for hosts with each symbiont, as these would be expected to fluctuate under thermal stress. Chaperones should respond by repairing damaged proteins while the immune genes may be activated to cope with shifts in symbiont behaviour or abundance (Mendiola et al. 2024). However, the general expression of the chaperone genes (vulnerable symbiont—df = 2, *F* = 0.02, *p* = 0.98; resistant—df = 2, *F* = 0.22, *p* = 0.80) and immune genes (vulnerable symbiont—df = 2, *F* = 0.66, *p* = 0.51; resistant—df = 2, *F* = 0.04, *p* = 0.96) remained the same across temperatures for hosts provided with each symbiont (Figures S2 and S3; Table S6). When evaluating for differential expression between the 24°C–36°C comparison group, two chaperones were differentially expressed, peptidyl‐prolyl cis‐trans isomerase‐like 2 protein and cathepsin L, partial protein which were down‐ and up‐regulated respectively, while only one immune gene was upregulated, ascorbate‐specific transmembrane electron transporter 1‐like protein.

### 3.4 Identification of Symbiont Genes Differentially Expressed With Temperature

**FIGURE 3:**
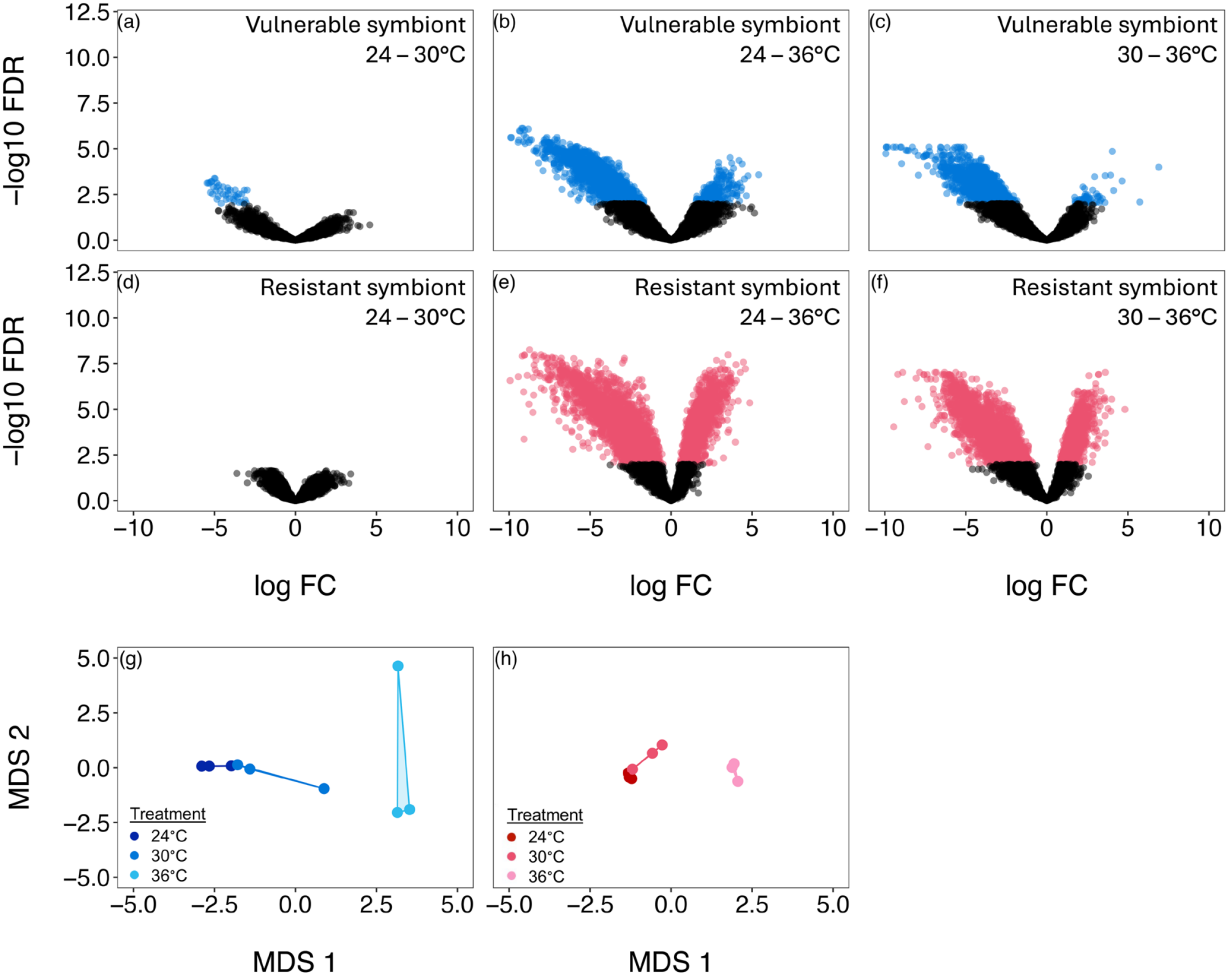
Transcriptional differences between thermal conditions are depicted with volcano plots, with coloured points indicating differentially expressed transcripts by pairwise comparison (FDR‐corrected *p* ≤ 0.01, greater than log2‐fold change ≥ |1|) for (a) vulnerable symbiont: 24°C–30°C, (b) vulnerable: 24°C–36°C, (c) vulnerable: 30°C–36°C, (d) resistant symbiont: 24°C–30°C, (e) resistant: 24°C–36°C, (f) resistant: 30°C–36°C. Multidimensional scaling showing ordination of replicates for each of the treatment groups (symbiont × temperature condition) with (g) vulnerable and (h) resistant symbionts.

After finding no variation in host transcription between symbiont treatment groups, we analysed the transcriptomes of each symbiont to identify the putative genes that may be associated with differential host outcomes under thermal stress. We evaluated the two *Caballeronia* symbiont transcriptomes separately as they are two distinct species. We first performed differential expression analysis (FDR corrected *p* < 0.01, with a log2‐fold change ≥ |1|). This revealed that both symbionts experienced a substantial downregulation of gene expression under thermal stress conditions with both the 24°C–36°C and the 30°C–36°C comparison groups each having about 1000 more genes downregulated at 36°C compared to those that were upregulated (Table 1). Furthermore, the vulnerable symbiont differentially expressed far fewer genes under thermal stress conditions compared to the resistant symbiont, with 31% and 57% fewer genes differentially expressed for the 24°C–36°C and the 30°C–36°C comparison groups, respectively (Figure 3, Table 1). Between the lower temperatures (24°C–30°C comparison), there were nearly no differentially expressed genes for either symbiont (Table 1).

**TABLE 1:** Number of differentially expressed genes in the symbionts between temperature treatments. Direction of differential expression is based on the first temperature relative to the second temperature. Host data are excluded as there were no differences between hosts with different symbiont strains.

	24°C–30°C		24°C–36°C		30°C–36°C	
	Up regulated	Down regulated	Up regulated	Down regulated	Up regulated	Down regulated
Vulnerable	0	42	213	1963	54	1233
Resistant	0	0	1026	2121	1035	1945

A rank‐based GO enrichment was performed individually on each symbiont species to evaluate changes in symbiont expression under thermal stress conditions. Rankings were based on a log2‐fold change ≥ |1| of symbiont genes. The goal was to summarise biological processes and to identify if certain processes were consistently represented between symbionts or between thermal stress comparison groups. The enrichment analysis further revealed similar responses to thermal stress between the 24°C–36°C and 30°C–36°C comparison groups within each symbiont (vulnerable symbiont: *r* ^2^ = 0.36, *p* < 0.001; resistant symbiont: *r* ^2^ = 0.71, *p* < 0.001), but there were stark differences in the functional enrichment between the two symbionts (*r* ^2^ = 0.03, *p* < 0.001; *r* ^2^ = 0.01, *p* = 0.09) (Figures S4 and S5). Under thermal stress, both symbionts showed enrichment of upregulated genes involved in ‘cell motility’ or ‘locomotion,’ and ‘protein folding’ and enrichment of downregulated genes associated with the ‘response to external stimuli.’ In the vulnerable symbiont, genes associated with ‘biological process involved in interspecies interaction between organisms’ and ‘exit from host cell’ were also enriched among downregulated transcripts.

### 3.5 Identification of Symbiont Heat Shock Associated Genes That Respond to Temperature

We hypothesised that expression profiles of heat shock genes would differ between the symbionts due to differences in their conferred host performance under thermal stress (Stillson et al. 2025) (Figure 1). To complement the genes identified through GO enrichment analysis, we compiled an additional literature‐based list of well‐characterised heat shock‐associated genes to ensure we captured key genes that span multiple GO categories (Roncarati and Scarlato 2017). Of the genes detected, only 12 would have been grouped solely under ‘protein folding’ (chaperones) and just 5 under ‘response to heat,’ while several, such as transcriptional regulators (e.g., *RpoH* , *HrcA* ), are distributed across broader stress‐response categories. By combining the GO enrichment and targeted literature‐based approaches, we identified 18 and 19 heat shock associated genes in the vulnerable and the resistant species respectively with copy numbers ranging from 1 to 7 for a total of 31 and 29 genes (Table S7). This approach allowed us to identify additional stress‐related genes that might have been overlooked by GO enrichment alone but are known to play roles in heat shock regulation.

**FIGURE 4:**
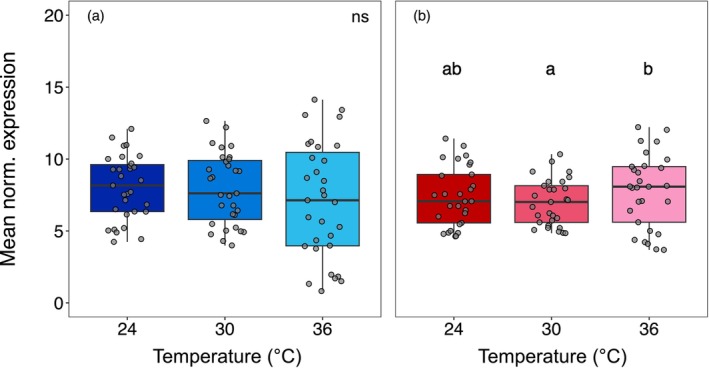
Mean normalised expression for the identified heat shock associated genes for each of the three temperatures in (a) vulnerable and (b) resistant symbiont species. Letters above bars denote statistically significant differences between temperatures (Tukey's HSD ≤ 0.05).

For the vulnerable symbiont, mean expression of heat shock genes did not differ significantly across temperatures (df = 2, *F* = 2.43, *p* = 0.09; Figure 4a). Differential expression analysis showed that in the 24°C–36°C comparison, four repair genes (*ClpB*, *DnaJ*, *DnaK*, *Lon*) were upregulated, while six transcriptional regulators were downregulated (*htrB*, two copies of *Hsp20*, three copies of *RpoE*). For the 30°C –36°C comparison, only *ClpB* was upregulated, with the same regulators downregulated except for *htrB*, which was not differentially expressed (Table S7, Figure S6). In the resistant symbiont, mean expression was lowest at 30°C and highest at 36°C (df = 2, *F* = 3.08, *p* = 0.05; Figure 4b). For the 24°C–36°C comparison, eight repair genes (*ClpB*, *ClpC*, *DnaJ*, *DnaK*, *GrpE*, *HslU*, *HtpG*, *Lon*) and three regulators (*HrcA*, *htrB*, *Hsp20*) were upregulated, while two repair genes (*htrA*, *Lon*) and two regulators (*Hsp20*, *RpoE*) were downregulated. In the 30°C–36°C comparison, expression profiles were similar except *ClpC* was not differentially expressed, while *FtsH*, *GroEL*, and the regulator *RpoH* were upregulated (Table S7, Figure S6).

### 3.6 Identification of Symbiont Motility Genes That Respond to Temperature

Symbiotic *Caballeronia* lose their flagellum and downregulate flagellum associated genes while inside their host (Ohbayashi et al. 2019). However, under thermal stress, these genes are overrepresented in both symbionts. Therefore, we further investigated the motility associated genes from the GO analysis, with 22 and 18 different flagellum associated genes identified in the vulnerable and resistant symbionts respectively (Table S7).

**FIGURE 5:**
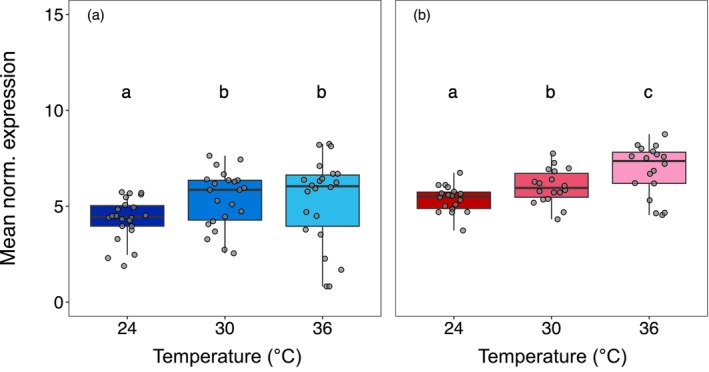
Mean normalised expression for the identified flagellar genes for each of the three temperatures in (a) vulnerable and (b) resistant symbiont species. Letters above bars denote statistically significant differences between temperatures (Tukey's HSD ≤ 0.05).

In the vulnerable symbiont, mean expression of these genes was lowest at 24°C and significantly higher at both 30°C and 36°C (df = 2, *F* = 5.69, *p* < 0.01; Figure 5a). Differential expression analysis showed that five genes were upregulated between 24°C and 36°C, while only one gene was downregulated between 30°C and 36°C (Figure S7). For the resistant symbiont, mean expression was lowest at 24°C and highest at 36°C (df = 2, *F* = 26.41, *p* < 0.001; Figure 5b). Here, 13 genes were upregulated and two were downregulated between 24°C and 36°C, while only one gene was downregulated between 30°C and 36°C.

## 4 Discussion

Our findings reveal that Eastern leaffooted bugs do not alter gene expression when provided with different symbiont species; rather, temperature‐dependent host outcomes may be driven by differences in gene expression profiles of *Caballeronia* symbionts under different temperature regimes. While we initially expected variation in the expression of symbiont heat shock genes between symbiont species, we observed a broader response, with the resistant symbiont not only showing a stronger reaction to heat stress compared to the vulnerable symbiont, but also increasing the expression of flagellar component genes. This suggests that the underlying mechanisms of temperature‐dependent symbiotic outcomes may include not only the ability of the symbiont to mount a heat‐shock response, but also unanticipated changes in symbiotic factors that have no obvious association with temperature (e.g., symbiont mobility). Investigating the link between symbiont gene expression and host performance is crucial in identifying how environmentally acquired microbial symbionts may contribute to host survival under stressful conditions, including the shifting extremes imposed by climate change.

### 4.1 Host Transcriptional Response

We expected that host transcription would vary between the symbionts, but our differential expression analysis detected no significant differences in host gene expression across symbiont treatments (this pattern persisted even when using a less stringent FDR < 0.05). However, we were unable to evaluate a full interaction model between symbiont identity and temperature. Thus, while we did not detect a main effect of symbiont on host transcription, we cannot rule out the possibility that hosts may exhibit symbiont‐specific and temperature‐dependent transcriptional responses. Future experiments are needed to fully assess whether such interactive effects occur.

Despite this limitation, our findings suggest that the variation in host outcomes under thermal stress may be driven by the differential symbiont responses to high temperature, rather than by changes in host gene expression. This work provides the first evidence that symbiont gene expression drives variation in host outcomes in the bug–*Caballeronia* symbiosis. Previous studies found that a single symbiont species (different from those studied here) confers inferior host performance under benign conditions (i.e., lower host weight and delayed development) (Hunter et al. 2022), but the mechanism behind this variation was not identified. All other *Caballeronia* rearing experiments have found that hosts receive similar benefits from any *Caballeronia* symbiont while reared under standard laboratory conditions (Acevedo et al. 2021; Hunter et al. 2022). However, once a stressor is applied, host outcomes can vary among symbionts (Stillson et al. 2025).

Lack of host transcriptional variation between symbionts may contribute to the flexibility observed in the bug‐*Caballeronia* system, in which hosts can utilise numerous symbiont strains and species with little to no symbiont specialisation (Stoy et al. 2023), although this flexibility can have drawbacks (Stillson et al. 2025). It is assumed that hosts associate with the first symbiont they find in their local environment, as the symbiotic organ seals shut 24 h after acquisition, preventing colonisation by subsequent microbes (Kikuchi et al. 2020). Furthermore, there is a priority effect, with the first symbiont acquired dominating the midgut crypts and any additional strains found at significantly lower abundances (Chen et al. 2024). Under benign conditions, symbiont strains may act similarly, but as environmental pressures change (i.e., thermal stress), different strains could benefit the host by buffering the insect from stressors, or the symbiont could fail its host and contribute to performance declines. This is observed in both corals and legumes in which thermally tolerant symbionts express higher levels of heat shock genes (including *DnaJ*, *DnaK*, and *GroESL*) when under thermal stress conditions compared to thermally sensitive strains (Alexandre and Oliveira 2011; Berkelmans and Van Oppen 2006).

### 4.2 Symbiont Transcriptional Response

The two symbionts varied in the number of heat shock genes they upregulated under thermal stress. Both symbionts upregulated transcripts for heat shock proteins *ClpB*, *DnaJ*, *DnaK*, and *Lon*. Of these, *ClpB*, *DnaJ*, and *DnaK* are bacterial chaperones that function in repairing proteins that have started to degrade due to high temperatures (Henderson et al. 2006), while *Lon* is a protease that functions in removing damaged proteins and polypeptides from the cell (Roncarati and Scarlato 2017). Beyond these four genes, the resistant symbiont upregulated regulatory proteins *HrcA*, *htrB*, and *Hsp20*, which contribute to the initiation of the heat shock cascade and activate various pathways in response to thermal stress (Roncarati and Scarlato 2017). The resistant symbiont also upregulates chaperones *ClpC* and *HtpG* and protease *HslU*, as well as *GrpE*, a cochaperone that works alongside *DnaJ* and *DnaK* to enhance their function (Harrison 2003; Henderson et al. 2006; Roncarati and Scarlato 2017). Together, these differentially expressed chaperones, proteases, and transcriptional regulators function as core components of the bacterial heat shock regulon and protein quality control pathways. Their coordinated upregulation suggests activation of the canonical bacterial stress response network, which works to prevent protein misfolding, degrade damaged proteins, and maintain cellular homeostasis under thermal stress (Roncarati and Scarlato 2017). This may suggest that the functional differences observed between these symbionts may be due to an increase in the expression of key thermal stress associated genes in the resistant symbiont. Failure to mount an appropriate heat shock response may have contributed to the vulnerable symbionts' thermal susceptibility, reducing its functionality and resulting in performance declines for the host when exposed to high temperatures (i.e., increased development time and increased mortality).

Reduction in symbiont heat shock gene expression has also been observed in both the vertically transmitted aphid symbiosis and other horizontally transmitted systems like legumes and corals and is associated with poor host outcomes. In aphids, if the symbiont expresses lower levels of heat shock gene *ibpA* under thermal stress conditions, its titre decreases, conferring worse host outcomes such as a decline in fecundity (Dunbar et al. 2007; Zhang et al. 2019). Interestingly, in bug‐*Caballeronia*, the symbiont titre remained relatively constant across temperatures, but the host experienced similar performance declines when temperatures increased (Stillson et al. 2025). In legumes, after experiencing high temperatures, thermally tolerant rhizobial symbionts increase the expression of heat shock genes *DnaK* and *GroESL* (Alexandre and Oliveira 2011). This increase in heat shock expression is essential for the maintenance of the symbiosis, as free‐living rhizobia cannot colonise their hosts if they are experiencing thermal stress (Alexandre and Oliveira 2013). Similarly, in coral algal symbionts, heat shock gene expression increases as part of the thermal stress response (Leggat et al. 2011), with some symbiont species being better able to tolerate high temperatures (Berkelmans and Van Oppen 2006), although the specific mechanisms behind the variation in coral thermal response are currently unknown. Taken together, these results highlight that the observed gene‐level differences likely reflect broader activation of conserved stress response pathways that enable some symbionts to maintain cellular function under heat stress, making horizontally acquired symbioses more resilient than vertically transmitted systems.

In addition to the increase in heat shock gene expression, the resistant symbiont also upregulated motility‐related genes. We found that 13/18 flagellar‐related genes (GO:0001539) were upregulated under thermal stress conditions in the resistant symbiont, while under benign conditions, previous work found 90% of flagellar synthesis genes are downregulated (Ohbayashi et al. 2019). This upregulation may suggest the beginnings of a mutualism breakdown, as the symbiont may be preparing to ‘escape’ the host. Activation of the flagellar genes appears to be a prerequisite for successful host evacuation (Xu et al. 2016), with flagellar genes reportedly only transcribed in the free‐living state (Ohbayashi et al. 2015, 2019). Therefore, the observed reactivation of flagellar genes may either be a direct response to the thermal stress or an indirect response to host stress (i.e., shifts in host immunity) making the gut inhospitable. Alternatively, the induction of motility may reflect symbiont movement within the gut to locate more favourable microhabitats under thermal and physiological stress conditions.

While our analyses reveal clear differences in transcriptional responses between symbionts, it is important to note that RNA expression levels do not always correlate with protein abundance or activity. Post‐transcriptional regulation, translational efficiency, and RNA and protein degradation rates can all influence functional outcomes. Therefore, although differential gene expression suggests mechanisms underlying thermal tolerance, further proteomic validation would strengthen these conclusions. Direct physiological assays, such as testing microbes harvested from the symbiont organ in soft agar motility assays or visualising flagella by microscopy, would help determine whether transcriptional activation of flagellar genes translates into functional motility under thermal stress conditions.

## 5 Conclusions

Our study suggests that variation in gene expression between *Caballeronia* species under thermal stress contributes to the differences in conferred host outcomes. At high temperatures, the resistant symbiont exhibited an increased level of heat shock gene expression and surprisingly also exhibited an increase in flagellar synthesis gene expression compared to the vulnerable symbiont. Many animals and plants associate with symbiotic bacteria that have a free‐living stage in the environment, but the bulk of research on temperature dependence in symbiotic outcomes has been conducted on vertically transmitted symbionts. Environmentally or horizontally acquired symbioses may operate differently than vertical symbioses, as there can be numerous symbiont species available in the environment, and the benefits conferred by these symbionts may depend on environmental conditions. With high symbiont variability, there is no guarantee that a host will acquire a beneficial symbiont strain, making environmental symbiont acquisition risky, but the ability of hosts to adjust to local environmental conditions by replacing their symbiont may help them better survive changing environmental pressures, benefitting the population as a whole.

## Author Contributions

P.T.S. and A.R. conceived the idea and designed the research. P.T.S. performed the research and collected data. S.B.S. and R.L.C. constructed and analysed host genome. P.T.S. analysed symbiont data. P.T.S. wrote the manuscript. P.T.S., S.B.S. and A.R. contributed to revisions. S.B.S. provided funding for the host genome sequencing. A.R. provided funding for the transcriptome sequencing.

## Conflicts of Interest

The authors declare no conflicts of interest.

## Supporting information


**Figure S1:** Hi‐C contact map of the assembled chromosomes. Nine autosomes and 1 X chromosome are highlighted in the large blue squares and are sorted according to their length. The smaller blue squares indicate the unplaced contigs. 0.00 MB 1714.55 MB assembly 0.00 MB 1714.55 MB.
**Figure S2:** Mean normalised expression for host chaperone genes for each of the three temperatures. Blues represent the vulnerable symbiont and reds represent the resistant symbiont. Statistical significance was evaluated using Tukey's HSD ≤ 0.05.
**Figure S3:** Mean normalised expression for host chaperone genes for each of the three temperatures. Blues represent the vulnerable symbiont and reds represent the resistant symbiont. Statistical significance was evaluated using Tukey's HSD ≤ 0.05.
**Figure S4:** Delta rankings for the selected enriched GO terms (biological process) identified using GO_MWU. Rankings are based on a log2‐fold change ≥ |1| of symbiont genes. Coloured cells show significantly enriched GO terms within each of the comparison groups (adjusted *p* ≤ 0.05).
**Figure S5:** Scatterplot of delta rank values from the GO MWU analysis (biological process) significant in both the (a) vulnerable symbiont treatments, (b) resistant symbiont treatments, (c) 24°C–36°C treatments, and (d) 30°C–36°C treatments when evaluating expression differences.
**Figure S6:** Mean normalised expression for the identified heat shock associated genes for each of the three temperatures in (a) V‐LZ003 and (b) R‐LZ019. Colours indicate the genes' roles in the heat shock response. Plots with multiple lines have multiple copies of the specified gene.
**Figure S7:** Mean normalised expression for the identified flagellar genes for each of the three temperatures in (a) V‐LZ003 and (b) R‐LZ019. Plots with multiple lines have multiple copies of the specified gene.

**Table S1:** Summary of *Leptoglossus phyllopus* assembly.
**Table S2:** Estimates of Leptoglossus phyllopus genome completeness.
**Table S3:** Estimates of Leptoglossus phyllopus genome annotation completeness.
**Table S4:** Summary of the RNA‐seq data.
**Table S5:** Number of differentially expressed genes in hosts between different treatments and with different symbiont strains. Direction of differential expression is based on the first treatment relative.
**Table S6:** Host chaperone and immune genes identified in Leptoglossus phylopus based on KO ID.

**Data S1:** Output files from Degust.

**Data S2:** Code used in analyses and figure generation.

## Data Availability

We deposited the HiFi and HiC reads associated with the genome assembly into the NCBI Sequence Read Archive (SRA) with the accession numbers SRR28382979 and SRR28382978 under BioProject accession number PRJNA1089323 and BioSample SAMN40534740. The whole genome assembly was assigned the NCBI genome accession number JBDNCC000000000. Transcript data were deposited into the NCBI SRA under BioProject PRJNA1089548. R code and the Degust output files are available in the supplement.
