# Supplementary material for: Symbiont Gene Expression Predicts Insect Host's Response to High Temperatures
Source: Mol Ecol. 2025 Oct 28;34(22):e70154. doi: 10.1111/mec.70154 (PMC12617022; doi:10.1111/mec.70154)

Title: Symbiont gene expression predicts insect host's response to high temperatures

Authors: Patrick T. Stillson, Sheina B. Sim, Renee L. Corpuz, and Alison Ravenscraft

Supplemental figures

Fig S1. Hi-C contact map of the assembled chromosomes. 9 autosomes and 1 X chromosome are highlighted in the large blue squares and are sorted according to their length. The smaller blue squares indicate the unplaced contigs.

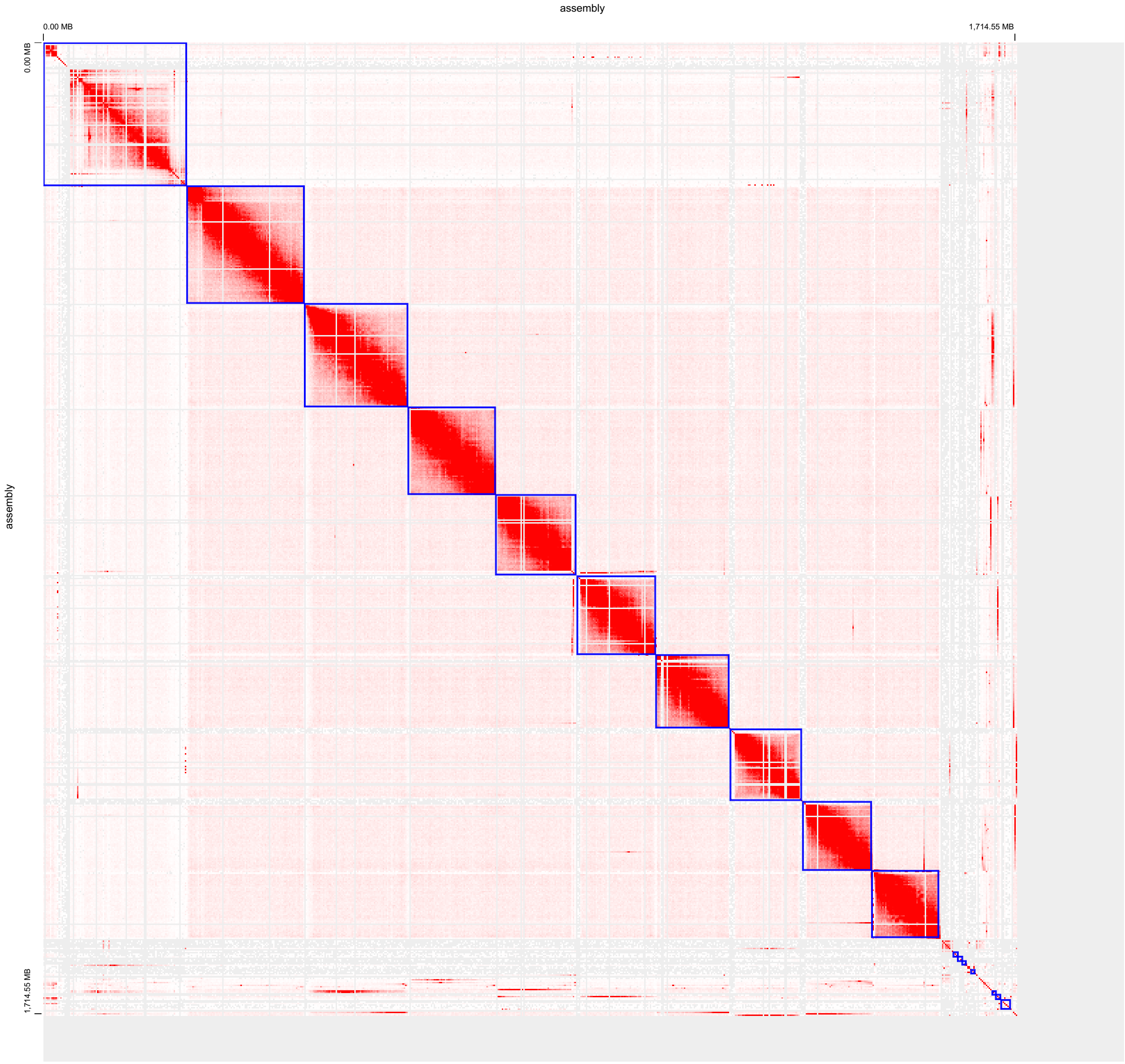

Fig S2. Mean normalized expression for host chaperone genes for each of the three temperatures. Blues represent the vulnerable symbiont and reds represent the resistant symbiont. Statistical significance was evaluated using Tukey's HSD ≤ 0.05.

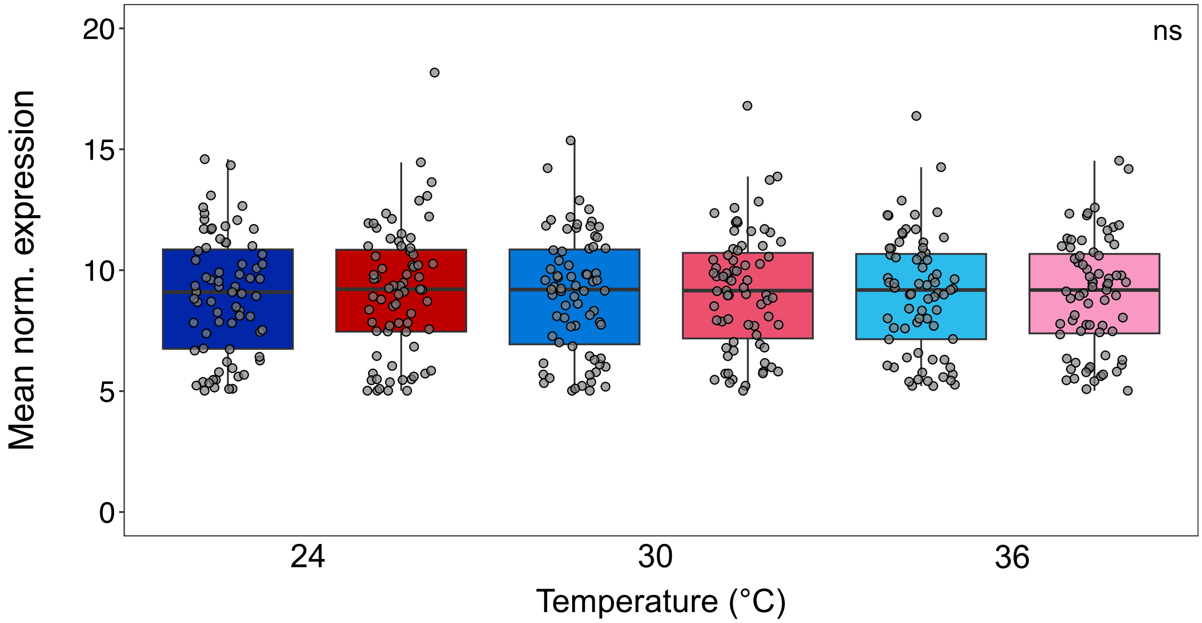

Fig S3. Mean normalized expression for host immune genes for each of the three temperatures. Blues represent the vulnerable symbiont and reds represent the resistant symbiont. Statistical significance was evaluated using Tukey's HSD ≤ 0.05.

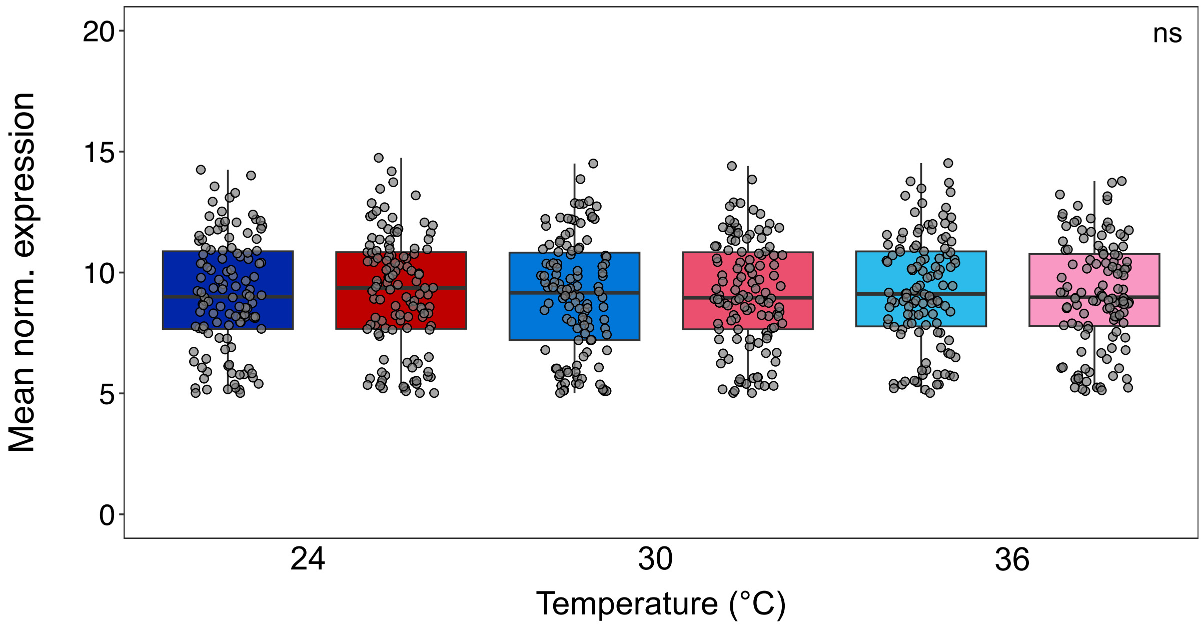

Fig S4. Delta rankings for the selected enriched GO terms (biological process) identified using GO_MWU. Rankings are based on a log2-fold change ≥ |1| of symbiont genes. Colored cells show significantly enriched GO terms within each of the comparison groups (adjusted $P \leq 0.05$)

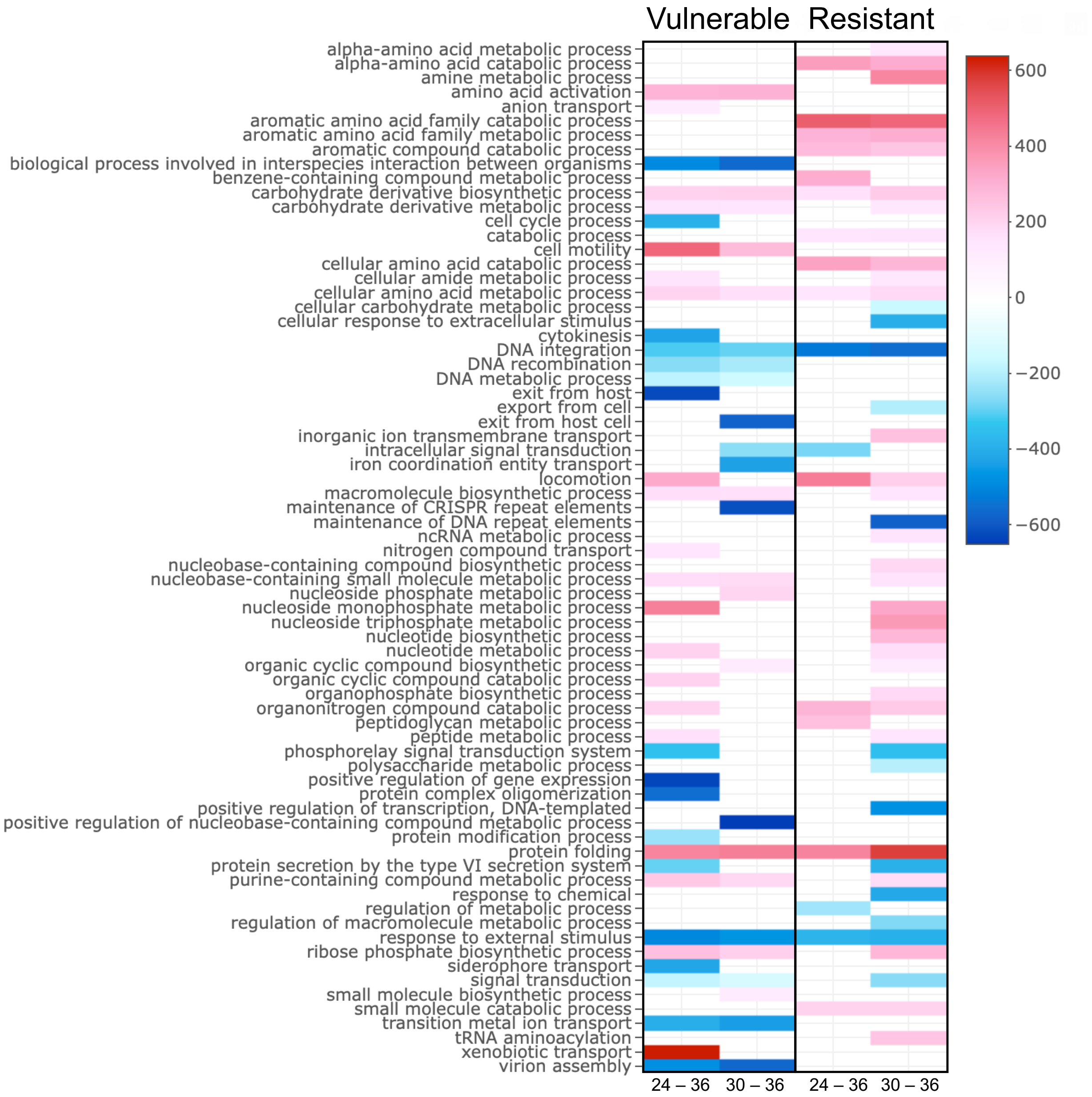

Fig S5. Scatterplot of delta rank values from the GO MWU analysis (biological process) significant in both the (a) vulnerable symbiont treatments, (b) resistant symbiont treatments, (c) 24 – 36°C treatments, and (d) 30 – 36°C treatments when evaluating expression differences.

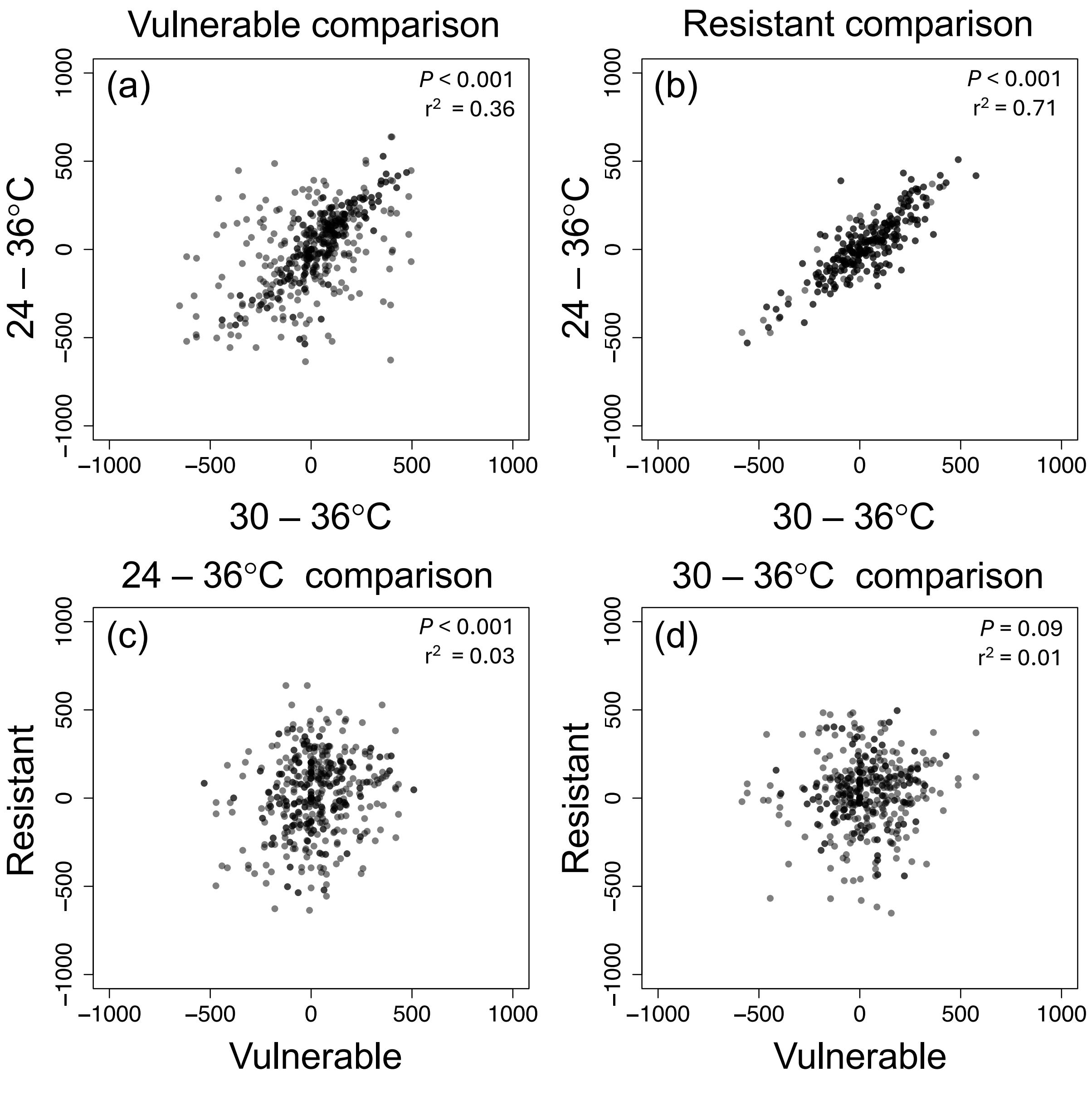

Fig S6. Mean normalized expression for the identified heat shock associated genes for each of the three temperatures in (a) V- LZ003 and (b) R-LZ019. Colors indicate the genes' roles in the heat shock response. Plots with multiple lines have multiple copies of the specified gene.

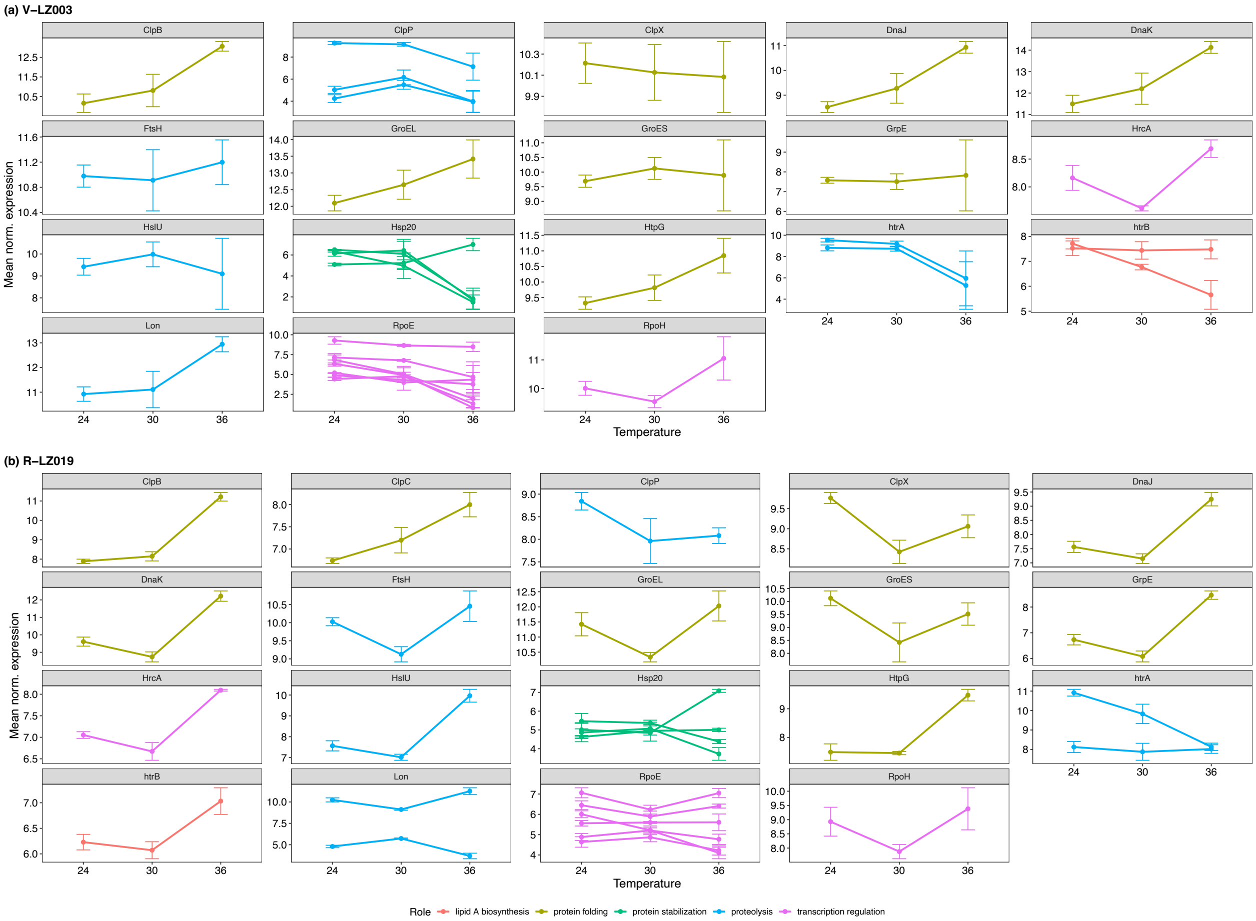

Fig S7. Mean normalized expression for the identified flagellar genes for each of the three temperatures in (a) V- LZ003 and (b) R-LZ019. Plots with multiple lines have multiple copies of the specified gene.

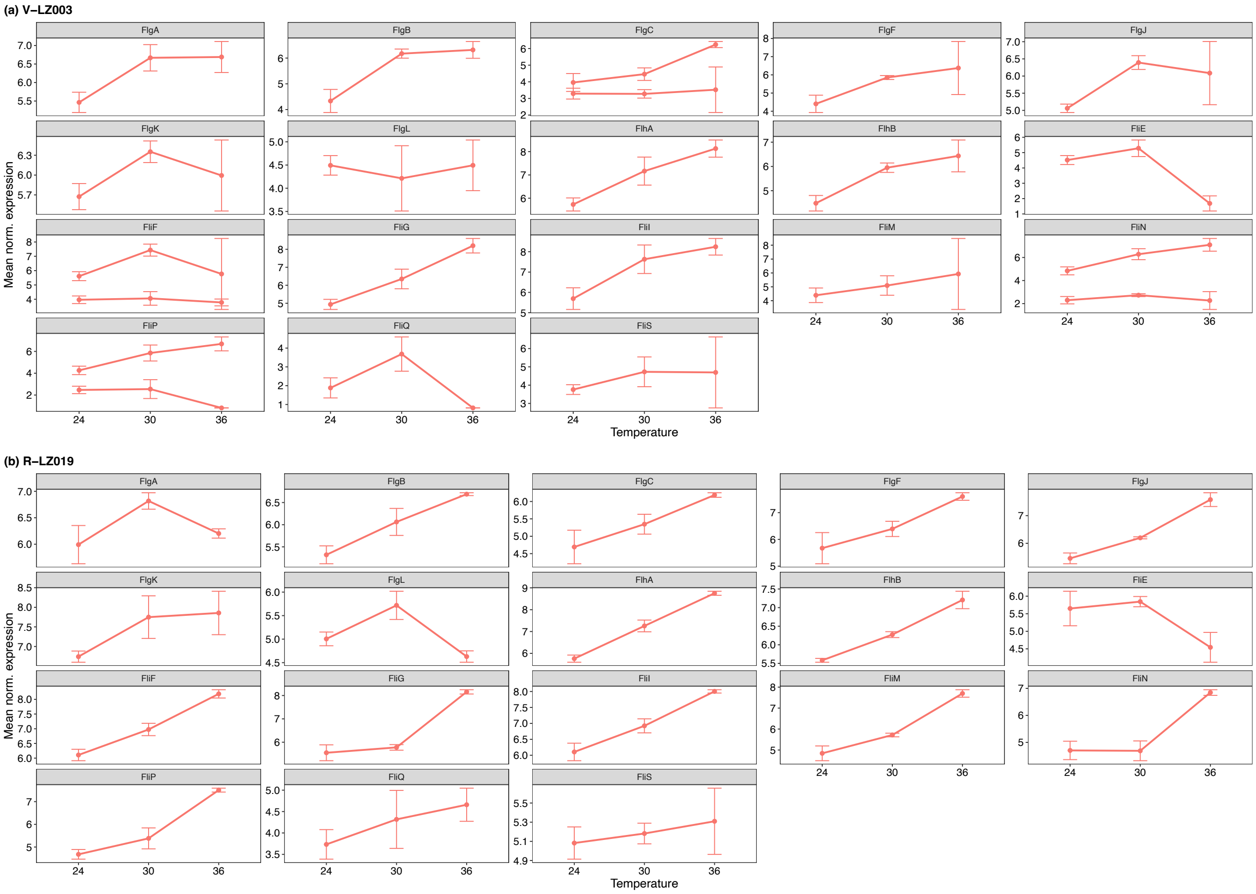

Supplement: Supplementary file 1 — Figure S1: Hi‐C contact map of the assembled chromosomes. Nine autosomes and 1 X chromosome are highlighted in the large blue squares and are sorted according to their length. The smaller blue squares indicate the unplaced contigs. 0.00 MB 1714.55 MB assembly 0.00 MB 1714.55 MB. Figure S2: Mean normalised expression for host chaperone genes for each of the three temperatures. Blues represent the vulnerable symbiont and reds represent the resistant symbiont. Statistical significance was evaluated using Tukey's HSD ≤ 0.05. Figure S3: Mean normalised expression for host chaperone genes for each of the three temperatures. Blues represent the vulnerable symbiont and reds represent the resistant symbiont. Statistical significance was evaluated using Tukey's HSD ≤ 0.05. Figure S4: Delta rankings for the selected enriched GO terms (biological process) identified using GO_MWU. Rankings are based on a log2‐fold change ≥ |1| of symbiont genes. Coloured cells show significantly enriched GO terms within each of the comparison groups (adjusted p ≤ 0.05). Figure S5: Scatterplot of delta rank values from the GO MWU analysis (biological process) significant in both the (a) vulnerable symbiont treatments, (b) resistant symbiont treatments, (c) 24°C–36°C treatments, and (d) 30°C–36°C treatments when evaluating expression differences. Figure S6: Mean normalised expression for the identified heat shock associated genes for each of the three temperatures in (a) V‐LZ003 and (b) R‐LZ019. Colours indicate the genes' roles in the heat shock response. Plots with multiple lines have multiple copies of the specified gene. Figure S7: Mean normalised expression for the identified flagellar genes for each of the three temperatures in (a) V‐LZ003 and (b) R‐LZ019. Plots with multiple lines have multiple copies of the specified gene. [file MEC-34-e70154-s001.pdf]
